# The power of local research to inform adverse childhood experiences in substance use prevention in adolescents and adults

**DOI:** 10.1186/s12889-022-13503-3

**Published:** 2022-06-15

**Authors:** Shiyou Wu, Sarah Lindstrom Johnson, Wendy Wolfersteig, Marisol Juarez Diaz, Maria Aguilar-Amaya

**Affiliations:** 1 School of Social Work, Arizona State University, Phoenix, USA; 2 The Sanford School of Social and Family Dynamics, Arizona State University, Phoenix, USA; 3 Southwest Interdisciplinary Research Center, Arizona State University, 400 E. Van Buren, Suite 800, Phoenix, AZ 85004 USA

**Keywords:** Adverse childhood experiences, Substance use, Prevention, Arizona youth survey, Behavioral risk factor surveillance system

## Abstract

**Background:**

The impact of adverse childhood experiences on substance use has been well reported, however, less well documented is looking at the comparison of youth and adult substance use and their respective adverse childhood experiences. This study leveraged local data sources to support prevention efforts inside a state-level working group and examined research questions that explored the relationship between reported adverse childhood experiences and substance use for youth and adult samples at the state level.

**Methods:**

This study conducted a series of logistic regression models (95% CI) between substance use outcomes with different age group populations to investigate the relationship between adverse childhood experiences and substance use for each group. Adverse childhood experiences scores and substance use were examined using two Arizona datasets: 1) Arizona Youth Survey (*n* = 42,009) and 2) the Behavioral Risk Factor Surveillance System (*n* = 5328).

**Results:**

The results of youth and adult datasets were consistent: users with adverse childhood experiences scores of 4 to 6 had a positive association with more substance use. When the variables were examined, showing the entire sample of youth and adult groups compared to those subgroups with a score of zero, a score of 1 to 3, and a score of 4 to 6, the overall pattern was the same; the more frequent use of substances was directly associated to the group with higher scores. Additionally, findings support increased attention on prevention and intervention efforts with higher reports of adverse childhood experiences as well as substance use.

**Conclusions:**

These findings demonstrate how local research can help prioritize prevention resources and increase the value of data-based decision-making. Policy-makers and providers can examine youth and adult data to compare priorities and assess for planning purposes. Specifically, it is possible to replicate known research findings, identify the most impacted subpopulations, and forecast the community’s future needs.

## Background

The consequences of adverse childhood experiences (ACEs) and toxic stress in adolescents and adults are public health crises in the United States. As studies validate the effects of ACEs on adult mental and physical health outcomes [1, 2] it is discouraging to know that in 2016, 34 million - nearly half of U.S. children (ages 0 to 17) - reported one ACE [3]. Further, one in ten children nationally has experienced three or more ACEs, placing them in a category of high risk [4]. In 2018, 61% of adults reported having experienced at least one ACE in their lifetime, and 16% had 4 or more ACEs [5]. Simultaneously, there has been an increase in U.S. adolescent and adult substance use [6]. This paper explores the relationship between ACEs and substance use for youth and adult samples and examines the associations of the youth compared to the adult findings.

### Adverse childhood experiences (ACEs) and substance use

ACEs refer to a series of stressful or traumatic events in childhood that have been associated with ongoing physical and social problems in adolescence and adulthood [7]. In the face of persistent and early life stress (such as ACEs), particular reward pathways in the brain (such as the hypothalamic-pituitary-adrenal axis) become dysregulated and do not function normally [8]. Human studies document a relationship between the neurobiological effects of child maltreatment and changes in the size and volume of areas of the brain associated with addiction, such as the prefrontal, thalamic, and cerebellar regions [9]. Post-traumatic stress symptoms have been shown to confer additional risk for substance use for both adults as well as young adults [10]. Additionally, studies suggest that early life stress is related to difficulties with emotion regulation, which is associated with substance use [11, 12].

Collectively, these pathways of association are reflected in the current literature connecting ACEs and substance use as well as associated mental health outcomes. The seminal work of Felitti et al. [1] demonstrated that retrospective report of ACEs in the first 18 years of life was related to a broad range of negative outcomes in adulthood, including smoking, depression, and suicidality. These findings have been replicated numerous times and extended to outcomes such as prescription drug misuse [13, 14] and intimate partner violence [15]. Studies of youth have found evidence for a gradient relationship between the number of ACEs and high-risk behaviors, including early adolescent (< 14) initiation of illicit drug use [16], smoking [13], and drinking alcohol [17]. Risk for substance use has been found for youth experiencing multiple types of victimization (e.g., child abuse, relationship violence; [18]). Exposure to violence also impacts youth mental health outcomes, increasing vulnerability to substance use behaviors [19].

### Prevention in the behavioral health system

Mental, emotional, and behavioral interventions operate across a continuum from promoting health behaviors to maintaining health behaviors, evidenced in the well-known model from the National Academies of Sciences, Engineering, and Medicine [20]. Critical for this paper are the most recent changes to emphasize the proportion of the model devoted to promotion and prevention, delineate promotion efforts to include society, community, and individuals and family, and incorporate a life-course perspective. ACEs represent an important mechanism to help state systems understand and prioritize promotion and prevention efforts. The awareness of ACEs and the negative relationship with health outcomes is widely known within both the medical community and the broader public [4, 21]. This has resulted in data being collected at state levels to understand individual, family, and even community adversity in early childhood. Inherently this represents both an understanding of social determinants of health as well as a life-course perspective. In fact, the prevention of ACEs was highlighted as an intervention with implications across the lifespan [20].

To help address this public health epidemic, the CDC advocates for upstream evidence-based prevention efforts such as collecting data on ACEs as a key risk factor. In 2019 the CDC supported six states to include an ACEs module in their BRFSS survey [22]. Finding this data of utmost importance, in 2020, the CDC decided to expand this by funding BRFSS and making the ACEs module available to all 50 states [22]. Similarly, many states and local- jurisdictions have modified their administration of the Youth Risk Behavior Survey (YRBS) or other local surveys to include an ACEs module. However, it is unclear how these data are used to guide state-level decisions regarding behavioral health resources, including prevention.

### The Arizona context

While investments to prevent disease and illness have proven to save lives, promote health equity, and be cost-effective [23], state prevention programs are profoundly underutilized and underfunded. Arizona is a notable example. In 2015 Arizona’s behavioral health system spent only 5% of its overall budget on prevention services [24]. This is despite the fact that Arizona has the highest number of children (ages 0–17) who have experienced two or more ACEs in the country, with a rate of 30% [25]. Additionally, in 2017 Arizona had the third-highest rate of drug overdose deaths in the West Census Region and the 24th highest overall in the country [22]. However, Arizona’s spending on opioid prevention only slightly increased from 22% in 2017 to 25% in 2018 [22].

Arizona has had a state-level data workgroup since 2004, which has regularly developed substance abuse profiles for use by agencies and policy decision-makers for programming and policy decisions. Multiple state agencies and other state entities that distribute and/or receive prevention, intervention and/or treatment funding have a representative, usually a data manager, as a member of this group. The pattern of examining substance use data continued with the workgroup undertaking this study using 2016 data for both youth and adults, with the added component of ACEs data to examine its impacts. As their agencies appoint workgroup members to participate in the workgroup, SAMHSA funding allocated for data work was used to contract a team of researchers (several of whom were workgroup members) to spend the time needed to conduct the analysis and provide results.

### This paper

This paper promotes leveraging sustainable data to support prevention. Specifically, this study analyzed ACEs and substance abuse data from adolescents using the Arizona Youth Survey (AYS; modeled after Communities that Care; [26]) and Arizona adults participating in the Behavioral Risk Factor Surveillance System (BRFSS) survey (CDC 2021 Congressional Justification, 2020). Statewide survey datasets from both youth and adult samples were analyzed to yield a comprehensive understanding of the relationship between reported ACEs and substance use. The inclusion of both populations allows for the understanding of current and future prevention and intervention needs as well as for a multi-generation perspective. As such, this paper illustrates the potential power of showing current needs among adults as well as forecasting future needs using adolescent data. Building clearly articulated prevention agendas driven by evidence in prevention science and well-integrated prevention infrastructures with increased capacities at the state level are critical, especially now due to the impacts and effects presented by the COVID pandemic and a growing awareness of structural inequalities [27]. Importantly, this analysis was commenced under the auspices of a state government committee comprised of researchers from multiple state agencies/entities. It, therefore, represents a systems-level model of leveraging data to encourage a focus on prevention. Implementation strategies call for collaboration and integration of perspectives to overcome implementation and dissemination obstacles [28]. These collaborations among researchers with consumers, practitioners, policymakers, and administrators can leverage their different perspectives to produce new knowledge. The literature on these types of partnerships is developing, and this paper contributes to this type of research-agency partnership that holds great promise for bridging the gap between research and practice. Specifically, we answer the following research questions: 1) what is the relationship between ACEs and substance use in Arizona youth and adult populations, and 2) what will similar examinations tell us about youth substance use and ACEs compared to adults.

## Methods

### Data and samples

Two Arizona datasets were identified that each had data on substance use and ACEs. In 2016, the Arizona Youth Survey (AYS; for youth population) and the Behavioral Risk Factor Surveillance System (BRFSS; for adult population) had collected primary data and were selected for analysis based on the similarity of their purposes, availability, and associated variables; only the similar variables were analyzed for this study.

**The Arizona Youth Survey (AYS)** is administered every 2 years (even years) to 8th, 10th, and 12th-grade students throughout all 15 counties in Arizona. It surveys Arizona students on risk and protective factors that affect the healthy development of children and adolescents and is designed to assess the prevalence and frequency of youth substance use and other risky behaviors in Arizona. All schools in Arizona are eligible to participate in the AYS, and efforts are made to ensure that data collected represents students across the state. The Arizona Criminal Justice Commission (ACJC) administers the survey with funds appropriated by the Arizona Legislature to comply with Arizona Revised Statute § 41–2416. Prior to the administration of the 2016 AYS survey, Arizona statute A.R.S. § 15–104 was amended, indicating that the ACJC can conduct the AYS without parental consent if the survey does not include depression or religiosity questions; further, the ACJC decided not to run afoul of A.R.S. § 15–102 which allows parents to object to any material about beliefs or practices in sex, morality or religion. Thus the four ACEs questions about sex/touching, depressed/mentally ill, and assessing child abuse and neglect were deemed inappropriate under Arizona statutes if the survey was to proceed without needing parental consent.

In 2016, the AYS was administered between February and May, with both paper and online modes used to allow flexibility for schools with limited student computer access. The 2016 AYS survey resulted in the participation of 57,170 students acquired from 249 schools across the 15 counties. AYS data are made available to schools and the public through the ACJC website (https://www.azcjc.gov/Programs/Statistical-Analysis-Center/Arizona-Youth-Survey).

**The Behavioral Risk Factor Surveillance System (BRFSS)** is a national system of health-related telephone surveys coordinated by the Centers for Disease Control and Prevention (CDC). States collect BRFSS data via a telephone survey to help them establish and track state and local health objectives, plan health programs, implement disease prevention and health promotion activities, monitor trends around chronic health conditions, and use of preventive services [5]. In Arizona, the BRFSS is managed through the Arizona Department of Health Services (ADHS), which collects data from randomly selected Arizona adults aged 18 and over living at home. In 2016, the state used a split survey design, whereby the core questions are asked of all respondents, and the added optional modules are split into two groups. This format allows for more optional questions to be added; the Adverse Childhood Experience module questions were asked only of the split 2 participants. Among the 10,952 eligible survey respondents in the Arizona total sample, 5328 adults (48.65%) completed the ACEs questionnaire portion of the phone interview. These results are generalizable to the extent that one considers the confidence intervals and the knowledge that, while not exact, these are the most reliable using national and valid state measures. The split sample of these data has more females and fewer Hispanics than the total sample of the Arizona population in 2016.

### Measures

#### Dependent variables: past 30-day substance use

Both datasets looked at the past 30-day substance use including: a) cigarette use (1/0); b) e-cigarette use (1/0); c) Had days with 1 or more drinks (1/0); d) Had binge drinking (1/0; Participants have X [X = 5 for men, X = 4 for women] or more drinks on occasion during the past 30 days =1; otherwise =0); and e) Illicit drug use (e.g., Marijuana, cocaine, meth, or heroin;1/0).

#### Independent variables: ACEs

The ACEs questions were adapted from those in the original ACEs study [1] but did not include questions on abuse or neglect. Participants were asked whether they had experienced: a) living with anyone who was a problem drinker or alcoholic; b) living with anyone who used illegal street drugs or who abused prescription medications; c) living with anyone who served time or was sentenced to serve time in a prison, jail, or other correctional facility; d) separated or divorced parents; e) adults in their home ever slap, hit, kick, punch, or beat each other up; and f) having an adult in their home ever swear at them, insult them, or put them down. The ACEs score was calculated using participants’ responses to the six different ACEs items. Based on the original ACEs study [1], which examined scores individually by ACEs scores of 0, 1, 2, 3, and 4 or more, three subgroups were created based on the total ACEs scores: 1) those who had a zero score; 2) those who scored between one and three, and 3) those participants who scored a four or higher.

#### Covariates

For demographic characteristics at the individual level, we included gender (1 = male; 0 = female); education (for AYS data, education was a categorical variable, and we created three dummy variables: grade 8 [1/0], grade 10 [1/0], and grade 12 [1/0, reference group]; for BRFSS sample, we treated education as a continuous variable); age (treated as a continuous variable); and race, which was indicated by selecting White (= reference group), Hispanic (1/0), Black or African American (1/0), Multi-race (1/0), and Other race (1 = Asian/Hawaiian/Native Americans; 0 = otherwise; only for BRFSS).

### Analytic strategies

Descriptive statistics were used to illustrate participant demographics to understand the characteristics of the AYS and BRFSS populations. Frequency counts were used to examine the participants’ reported gender, race, and education level. To understand the extent to which ACEs and substance use outcomes are related, a series of logistic regression models were conducted to examine the relationship between each of the substance use outcomes (dummy coded as 0/1) and the ACEs groups while controlling for the demographic variables (i.e., age, gender, education, race/ethnicity). Logistic regression is a form of predictive analysis and is used to describe the relationship between a dependent binary and the independent variables [14]. In addition, we tested the assumptions of the logistic regression models. For example, we calculated the variance inflation factor (VIF) to detect the multicollinearity issue. VIF values for model variables were less than 2, indicating the absence of multicollinearity. Listwise deletion approach was used to handle the missing data. We checked differences in the substance use outcome variables between those who remained in the analytic sample and those who were excluded, and had not observed statistically significant differences. Stata 15.0 was used to conduct the data analyses.

## Results

### Findings from the AYS youth sample

#### AYS demographics

The overall AYS sample characteristics and each of the three ACEs score subgroups (i.e., 0, 1–3, and 4–6) are shown in Table 1. The final sample was comprised of 42,009 respondents, with more females than males (51.51 and 48.62%, respectively). Among the full sample, within the past 30 days, 8954 (21.31%) have smoked, and 8287 (19.73%) have used illicit drugs, and 17,688 (42.06%) have used alcohol.

**Table 1:** AYS sample characteristics by ACEs score

	Total Sample	ACEs 0	ACEs 1–3	ACEs 4–6
	N; %/ Mean (*SD*)	N; %/ Mean (*SD*)	N; %/ Mean (*SD*)	N; %/ Mean (*SD*)
Sample size	42,009; 100.00	11,407; 27.00	24,003; 57.00	6599; 16.00
**Dependent Variables**				
Past 30-day use (1/0)				
**Smoking**	8954; 21.31	1106; 9.83	5155; 21.86	2693; 41.46
** E-cig.**	3723; 8.86	405; 3.61	2113; 8.97	1205; 18.55
**Illicit drugs**	8287; 19.73	1239; 10.86	4825; 20.10	2223; 33.69
**Alcohol**	17,668; 42.06	2863; 25.73	10,710; 45.70	4095; 63.37
**Binge drinking**	5264; 12.53	774; 6.91	3091; 13.16	1399; 21.62
**Sample characteristics by ACEs score**				
**Age**	15.36(1.17)	15.26 (1.71)	15.45 (17.44)	15.52 (1.66)
Gender				
**Female**	21,296; 51.51	5175; 45.92	12,342; 52.24	3779; 58.10
**Male**	20,103; 48.62	6094; 54.07	11,284; 47.76	2725; 40.64
Race				
**White**	18,815; 44.79	5275; 46.86	10,770; 45.34	2770; 42.40
**Black or African American**	1380; 3.29	339; 3.01	836; 3.52	205; 3.14
**Multiracial**	2279; 5.43	489, 4.34	1339, 5.64	451, 6.90
**Hispanic**	16,335; 38.88	4408;39.15	9249;38.94	2678;40.98
Education Level				
**8th Grade**	15,989; 38.06	4625; 40.55	9077; 37.82	2287; 34.66
**10th Grade**	14,029; 33.40	3644; 31.95	8100; 33.75	2285; 34.63
**12th Grade**	11,991; 28.54	3138; 27.51	6826; 28.44	2027; 30.72

About 27% (*n* = 11,407) of participants reported an ACEs score of zero. In this zero ACEs group, 25.73% drank alcohol, 10.86% used illicit drugs, 9.83% smoked, 6.91% reported binge drinking, and 3.61% reported the use of e-cigarettes. The subgroup with an ACEs score between 1 and 3 (57%) was made up of more females (52.24%) than males (47.76%); 45.34% of this subsample was White, followed by 38.94% Hispanic. By education level, 8th graders had the highest percentage of respondents reporting 1–3 ACEs at 37.82%. Past 30-day alcohol use for this ACEs 1–3 group was 45.70, 21.86% smoked, and 20.10% reported illicit drug use. For those with 4–6 ACEs (16%), 58.10% were female and 40.64% male. Again, the majority identified as White (42.40%), followed by Hispanic (40.98%). Eighth and 10th grade students made up equal shares (35%), reporting 4–6 ACEs. Thirty-day use was also greater than in the previous group, with 63.37% reporting alcohol use, 41.46% reporting smoking, and 33.69% reporting illicit drug use.

Table 2 shows results for the five substance use outcome variables, controlling for demographics variables. Compared to participants with no ACEs, those who have an ACEs score between 1 and 3 had significantly higher odds of using cigarettes (OR = 2.46, *p* < 0.001) and e-cigarettes (OR = 2.04, *p* < 0.001), and having days with one or more drinks (OR = 1.87, *p* < 0.001), binge drinking (OR = 1.88, *p* < 0.001), and using illicit drugs (OR = 2.58, *p* < 0.001). Similarly, compared to those who have no ACEs, results showed that those with an ACEs score of 4 or higher had greater odds of using cigarettes (OR = 7.02, *p* < .001), e-cigarettes (OR = 3.77, *p* < .001), and having days with one or more drinks (OR = 3.77, *p* < 0.001), binge drinking (OR = 3.90, *p* < 0.001), and using illicit drugs (OR = 6.67, *p* < 0.001).

**Table 2:** AYS regression results of past 30-day substance use on ACEs

AYS Logistic Regression Results of Past 30-day Substances Use on ACEs					
	Cigarettes	E-cigarettes	Days with 1 or more drinks	Binge Drinking	Illicit Drugs
	OR [95% CI]	OR [95% CI]	OR [95% CI]	OR [95% CI]	OR [95% CI]
**ACEs (ref. = 0 ACEs)**					
1–3 ACEs	2.46***	2.04***	1.87***	1.88***	2.58***
	[2.26–2.69]	[1.87–2.23]	[1.68–2.08}	[1.71–2.08]	[2.33–2.85]
4–6 ACEs	7.02***	3.77***	3.77***	3.90***	6.67***
	[6.37–7.73]	[3.40–4.17]	[3.35–4.25]	[3.49–4.34]	[5.97–7.45]
**Covariates**					
** Age**	1.37***	1.21***	1.41***	1.38***	1.24***
	[1.31–1.43]	[1.16–1.27]	[1.33–1.49]	[1.31–1.45]	[1.18–1.30]
** Male**	0.97	0.73***	0.93	1.01	0.86***
	[0.91–1.03]	[0.68–0.78]	[0.86–1.00]	[0.94–1.08]	[0.81–0.92]
**Education levels** (ref. = Grade 12)					
Grade 8	1.09	1.06	0.70**	0.81	0.54***
	[0.91–1.31]	[0.87–1.29]	[0.55–0.89]	[0.66–1.01]	[0.44–0.67]
Grade 10	1.09	1.12	1.04	1.02	0.88*
	[0.97–1.22]	[0.99–1.29]	[0.90–1.20]	[0.90–1.17]	[0.77–1.00]
**Race** (ref. = White)					
Black	0.77*	0.50***	0.72*	0.78*	0.99
	[0.63–0.95]	[0.96–1.10]	[0.55–0.94]	[0.61–0.99]	[0.80–1.22]
Multiracial	1.24**	0.88	0.93	0.92	1.17*
	[1.08–1.42]	[1.87–2.23]	[0.78–1.11]	[0.78–1.09]	[1.01–1.36]
Hispanic	1.45***	1.03	1.09*	1.23***	1.22***
	[1.36–1.55]	[0.96–1.10]	[1.01–1.19]	[1.14–1.33]	[1.14–1.31]
Constant	0.001***	0.006***	0.000***	0.000***	0.003***
Observations	31,253	31,932	32,028	31,879	32,143

Note. * *p* < .05, ** *p* < .01, *** *p* < .001; *OR*odds ratios; *95%CI* 95% Confidence intervals; *ref.* reference group

Table 1 shows results for the five substance use outcome variables, controlling for demographics variables. Compared to participants with no ACEs, those who have an ACEs score between 1 and 3 had significantly higher odds of using cigarettes (OR = 2.46, *p* < 0.001) and e-cigarettes (OR = 2.04, *p* < 0.001), and having days with one or more drinks (OR = 1.87, *p* < 0.001), binge drinking (OR = 1.88, *p* < 0.001), and using illicit drugs (OR = 2.58, *p* < 0.001). Similarly, compared to those who have no ACEs, results showed that those with an ACEs score of 4 or higher had greater odds of using cigarettes (OR = 7.02, *p* < .001), e-cigarettes (OR = 3.77, *p* < .001), and having days with one or more drinks (OR = 3.77, *p* < 0.001), binge drinking (OR = 3.90, *p* < 0.001), and using illicit drugs (OR = 6.67, *p* < 0.001).

Table 2 results also show that for each 1 year increase in age, the odds increase for each of the five substance use outcomes (OR ranges between 1.21 and 1.41; *p* < 0.001). Compared to females, males reported significantly lower odds of e-cigarette use (OR = 1.27, *p* < 0.001) and illicit drug use (OR = 1.13, *p* < 0.001). In addition, compared with youth from Grade 12, students from Grade 8 had significantly lower odds of substance use for having days with one or more drinks and using an illicit drug, and students from Grade 10 had significantly lower odds of substance use for using an illicit drug in the past 30 days (OR = 1.12, *p* < 0.001). Regarding race and ethnicity, the results showed significant differences in substance use between White and Black participants, with lower odds of using among Black participants (OR ranges between 0.50 and 0.78; *p* < .05) for cigarettes and alcohol uses in the past 30 days. However, compared with Whites, Hispanic participants had higher odds of using all five substances (OR ranges between 1.03 and 1.45; all *p* values < .05 except for using e-cigarettes).

### Findings from the BRFSS adult sample

#### BRFSS demographics

The overall BRFSS sample characteristics and results by each of the three ACEs score subgroups (i.e., 0, 1–3, and 4–6) are shown in Table 3. The final sample was comprised of 5328 respondents, with more females than males (58.60 and 41.40%, respectively). Among the full sample, 160 (3.00%) have used an e-cigarette, 134 (2.52%) have used illicit drugs, and 2545 (48.21%) have used alcohol in the past 30-days.

**Table 3:** BRFSS sample characteristics by ACEs score

	Total Sample	ACEs 0	ACEs 1–3	ACEs 4–6
	N; %/ Mean (*SD*)	N; %/ Mean (*SD*)	N; %/ Mean (*SD*)	N; %/ Mean (*SD*)
Sample Size	5328; 100.00	727; 14.00	4296; 81.00	305; 5.00
**Dependent Variables**				
Past 30-day use (1/0)				
**Smoking**	621; 11.66	64; 8.82	481; 11.20	76; 24.92
**E-Cig.**	160; 3.00	13; 1.79	121; 2.82	26; 8.52
**Illicit Drugs**	134; 2.52	5; 0.69	101; 2.35	28; 9.18
**Alcohol**	2545; 48.21	244; 34.27	2146; 50.34	155; 50.99
**Binge Drinking**	439; 8.29	45; 6.28	348; 8.14	46; 15.08
**Sample characteristics by ACEs score**				
**Age**	60.15(17.97)	57.39 (19.86)	61.16 (17.44)	52.46 (18.03)
Gender				
**Female**	3122; 58.60	391; 53.78	2555; 59.47	176; 57.70
**Male**	2206; 41.40	336; 46.22	1741; 40.53	129; 42.30
Race				
**White**	3980; 74.70	454; 62.45	3312; 77.09	214; 70.16
**Black Or African American**	114; 2.14	17; 2.34	85; 1.98	12; 3.93
**Other**	513; 9.63	127; 17.47	355; 8.22	33; 10.82
**Multiracial**	66; 1.24	15; 2.06	45; 1.05	6; 1.97
**Hispanic**	655; 12.29	114; 15.68	501; 11.66	40; 13.11
Education Level				
**Primary Or Less**	130; 2.44	29; 3.99	97; 2.26	4; 1.31
**Some High School**	230; 4.32	40; 5.50	175; 4.07	15; 4.92
**High School Graduation**	1258; 23.61	193; 26.55	973; 22.65	92; 30.16
**Some College Or Tech School**	1585; 29.75	212; 29.16	1277; 29.73	96; 31.48
**College Graduation**	2125; 39.88	253; 34.80	1774; 41.29	98; 32.13

About 14% (*n* = 727) of participants reported an ACEs score of zero, of which about a third (34.27%) drank alcohol. For the participants who reported an ACEs score of 1–3, slightly over half drank alcohol (50.34%), while 11.20% smoked, 8.14% reported binge drinking, and less than 3% reported using e-cigarettes or illicit drugs. The subgroup with an ACEs score between 1 and 3 (81%) was made up of more females (59.47%) than males (40.53%); 77.09% of this subsample was White, followed by 11.66% Hispanic. Participants with a college degree had the highest percentage of respondents reporting 1–3 ACEs at 41.29%. In the last subgroup were those with 4–6 ACEs (5%); 57.7% of this group was female. For this group, about 51% drank alcohol, 24.92% reported having smoked, 15.08% reported binge drinking, 9.18% used illicit drugs, and 8.52% used an e-cigarette in the past 30-days. Further, about 70% of this group was White and 13% Hispanic, with about 30% in each education group being a high school graduate, having some college, or being a college graduate.

### BRFSS regression results of past 30-day substance use on ACEs

Table 4 shows that, all other variables held constant, compared to participants with no ACEs, those who have an ACEs score between 1 and 3 had significantly higher odds of having days with one or more drinks (OR = 1.82, *p* < .001), binge drinking (OR = 1.56, *p* < .01), and using illicit drugs (OR = 3.93, *p* < .01). In addition, compared to participants with no ACEs, those who have an ACEs score between 1 and 3 had significantly higher odds of using cigarettes (OR = 1.49, *p* < .01) and e-cigarettes (OR = 1.80, *p* < .05).

**Table 4:** BRFSS logistic regression results of past 30-day substance use on ACEs

VARIABLES	Cigarettes	E-cigarettes	Days with 1 or more drinks	Binge Drinking	Illicit Drugs
	OR [95% CI]	OR [95% CI]	OR [95% CI]	OR [95% CI]	OR [95% CI]
**ACEs** (ref. = 0 ACEs)					
1–3 ACEs	1.49**	1.80*	1.82***	1.56**	3.93**
	[1.13–1.98]	[1.00–3.23]	[1.53–2.16]	[1.12–2.17]	[1.59–9.76]
4–6 ACEs	3.25***	4.29***	1.94***	2.42***	12.82***
	[2.24–4.74]	[2.15–8.56]	[1.46–2.57]	[1.55–3.79]	[4.85–33.90]
**Covariates**					
**Age**	0.98***	0.97***	0.99**	0.97***	0.97***
	[0.98–0.99]	[0.96–0.97]	[0.99–1.00]	[0.96–0.97]	[0.96–0.97]
**Male**	1.25*	1.23	1.51***	2.32***	2.66***
	[1.05–1.48]	[0.89–1.69]	[1.35–1.69]	[1.89–2.85]	[1.85–3.84]
**Education levels**	0.65***	0.79**	1.47***	1.04	1.06
	[0.60–0.71]	[0.68–0.93]	[1.39–1.56]	[0.93–1.15]	[0.88–1.28]
**Race** (ref. = White)					
Black	1.10	0.90	1.01	1.21	0.85
	[0.65–1.85]	[0.35–2.30]	[0.69–1.49]	[0.67–2.21]	[0.30–2.43]
Other	0.74	0.51*	0.47***	0.67*	0.57
	[0.55–1.00]	[0.28–0.92]	[0.38–0.58]	[0.47–0.95]	[0.31–1.06]
Multiracial	1.74	0.64	0.67	0.85	1.62
	[0.93–3.27]	[0.15–2.76]	[0.40–1.11]	[0.35–2.05]	[0.55–4.80]
Hispanic	0.57***	0.47**	0.75**	0.95	0.23***
	[0.43–0.76]	[0.27–0.81]	[0.63–0.91]	[0.70–1.29]	[0.10–0.55]
Observations	5326	5326	5132	5279	5296

Note. * *p* < .05, ** *p* < .01, *** *p* < .001; *OR* odds ratios; *95%CI* 95% Confidence intervals; *ref.* reference group

Similarly, compared to participants with no ACEs, those who have an ACEs score of 4–6 had significantly higher odds of having had days with one or more drinks (OR = 1.94, *p* < .001), binge drinking (OR = 2.42, *p* < .001), and used illicit drugs (OR = 12.82, *p* < .001). Results further showed that those with an ACEs score of 4 or higher had significantly higher odds of using cigarettes (OR = 3.25, *p* < .001) and e-cigarettes (OR = 4.29, *p* < .001).

Table 4 results also showed that every 1 year increase of age significantly decreased the odds of using each of the five substance use outcomes (OR ranges between 0.97 and 0.99; *p* < .01). Compared to females, males reported higher odds of using all five substances (OR ranges between 1.23 and 2.66; all *p* values < .05 except for using e-cigarettes). Every one increased level of education significantly decreased the odds of using cigarettes (OR = 0.65, *p* < .001) and e-cigarettes (OR = 0.79, *p* < .01), but significantly increased the odds of having days drinking 1 or more drinks during the last 30 days (OR = 1.47, *p* < .001). In terms of race and ethnicity, the results showed that there is no significant difference in substance use between White and Black participants; however, compared with Whites, Hispanic participants had significantly lower odds of using all five substances (OR ranges between 0.23 and 0.95, all *p* values < .05 except for binge drinking).

## Discussion

This study conducted a series of similar statistical analyses with two state-level datasets of different age group populations to examine the degree of relationships between ACEs and substance use for each group. We found for the youth sample, compared with those who had an ACEs score of 1 to 3, those who had an ACEs score of 4 to 6, had significantly higher odds ratios, and the 95% confidence intervals did not have overlaps for all the five outcome variables, indicate clear graded relationships between experience of childhood adversity and all substance use outcomes. Although the adult sample showed a similar trend of the group differences that those who had an ACEs score of 4 to 6, had significantly higher odds ratios than those who had an ACES score of 1 to 3, there are some overlaps of the 95% confidence intervals. The unclear graded relationship among the adult sample might be because of the larger variation and age range of the sample than the youth sample. However, findings on ACEs in Arizona have not previously been examined in a manner that allowed for youth and adult comparisons of findings. While all ACEs are key factors, these Arizona percentages of youth and adults who have 4–6 without the inclusion of the other four ACE variables, show that ACEs are extremely important to consider when examining substance use.

These findings complement and extend existing work that has studied these populations and outcomes separately [29]. Further, the National Academies of Sciences, Engineering, and Medicine model with its continuum of health behaviors has an updated emphasis on promotion and prevention, which, along with a focus on research-agency partnerships around evidence-based interventions, represents a movement toward leveraging data at a systems-level to focus on prevention [20]. This paper highlights how using both youth and adult data-sets can help state agencies understand both current needs as well as predict future need.

A prevention agenda guided by data can help address the public health epidemic identified by the CDC stemming from the impact of ACEs on mental and physical health. Most studies have focused only on limited substance use outcomes or only one population in analyzing how ACEs influence these outcomes. Uniquely, this study both 1) examined the association between ACEs and multiple substance use outcomes, and 2) conducted analyses with both youth and adult samples, which can benefit a systems-level approach to prevention. The CDC continues to support state-level data collection for adults (BRFSS) and youth (YRBS), providing states and local jurisdictions with the opportunity to conduct similar comparisons across datasets. By understanding the similarities and differences of the findings between the youth (AYS) and adult (BRFSS) sample populations, insights can be gained vital to data-driven programming and policies that can help decrease unwanted substance use behaviors. Additionally, by examining both data sets insight is gained as to how ACEs impacts substance use not only during adolescence but well into adulthood, indicating the importance of prevention early in a child’s life. The findings of this study may serve as a reference for other states that have substance use and ACEs data that are publicly available in order to conduct state-level comparisons to Arizona.

### Similarities and differences for youth and adult samples

When the ACEs and substance use variables were examined showing the entire sample of youth (AYS) and adult (BRFSS) groups compared to those subgroups with an ACEs score of zero, an ACEs score of 1 to 3, and an ACEs score of 4 to 6, the overall pattern was the same: the more frequent use of substances was directly associated to the group with more ACEs (Tables 1 and 2). Shin and colleagues [30] found that youth experiencing high/multiple ACEs had a higher likelihood of alcohol problems and current tobacco use. Choi and colleagues [29] similarly found an association between ACEs and lifetime substance use disorders (alcohol, nicotine, or drug), but primarily for experiences of abuse or living with a parent with substance use or mental health problem. Examining substance use and ACEs together has sought to indicate the extent of the overlap of these problem indicators and the persistent negative effects on health over time, prioritizing prevention and early intervention. Identifying these areas of need by using data is important in determining where to spend prevention and treatment resources for states such as Arizona, which, according to the United Health Foundation (UHF) [31], ranks #46 for state dollars that are dedicated to public health, with a value of an estimated $57 per person in comparison to the average estimated average value of $173.70 for the top ten states in the U.S.

It is possible to identify specific population groups and needs for substance- and ACEs-targeted evidence-based programming from examining these data. Arizona’s future adults (i.e. youth sample) are already reflecting the increased need for substance use prevention and intervention programs based upon higher percentages of use by youth than by adults. For example, Arizona female youth may require attention as the percentage with 4–6 ACEs was higher than the overall youth percentage, while the percentage of adult females with an ACEs score of 4 to 6 was approximately the same as the percentage of females in the sample overall. However, this is complicated by research suggesting a stronger relationship between ACEs and substance use outcomes for men [29] and supports further attention to understanding mechanisms through which ACEs impact substance use [32]. As to racial/ethnic implications of youth to adult findings, compared with Whites, Hispanic youth participants had greater odds of using all five substances (although not significant for the e-cigarettes); however, compared with Whites, Hispanic adult participants had lower odds of using all five substances (although not significant for binge drinking) during the last 30 days. Additionally, the youth sample had 20% more Hispanic respondents and 30% fewer White respondents than did the adult sample, and this gap existed by race across all categories of ACEs scores; this seemingly reflects the changing demographics in Arizona toward a majority-minority population. Addressing the needs around substance use amongst Hispanic youth is becoming an ongoing concern and area for intervention as Hispanics will continue to be the largest ethnic minority group in the United States [14]. Additionally, research examing the influence of ACEs on drinking outcomes identified the highest risk of heavy drinking for adult Hispanics experiencing both household challenges and abuse [33]. Given changes in both demographic populations as well as the immigration context and the unfortunate reality that youth of color experience more ACEs, racial/ethnic differences in the impact of ACEs on substance use need additional research.

Not surprisingly, alcohol continues to be the 30-day drug of choice for both youth and adults. Adults reported a higher percentage of alcohol use overall than youth with almost half of the adults and 4 in 10 youth who had consumed alcohol in the past 30 days. For those with an ACEs score of 4 to 6, almost two-thirds of youth and half of adults reported regular alcohol use. As to binge drinking in the past 30 days, for those with an ACEs score of 4 to 6, 1 in 5 youth and just slightly fewer adults reported this high-risk behavior. Although it has long been easy for youth to obtain alcohol, it is illegal for those under 21 years of age in Arizona. Given these high percentages who use, we cannot ignore alcohol as both a gateway and long-term drug that needs attention in prevention, intervention, treatment, and trauma-informed planning and policies.

### Benefits of systems collaboration (implications)

The 2017 Kids Count Report ranked Arizona #46 compared to other states in overall child well-being [34]. Arizonan policymakers could improve these rankings using substance use and ACEs data to understand the situation better and direct its prevention efforts. The collection of AYS and BRFSS data is regular and ongoing, and the capability to compare the results on both substance use and ACEs data has now been demonstrated. The ability to use these data within state and county agencies, the statewide workgroup, the university, and other research settings should be used more extensively for needs assessments, program planning, priority setting, resource allocation, and evaluation, including examining year-over-year trends. For example, Alaska, California, and Tennessee used BRFSS ACEs data to examine the degree of ACEs and how ACEs contribute to chronic health conditions and costs in their states. “This type of analysis provides information on the cost burden of childhood adversity and the potential savings of preventing childhood trauma and related chronic health conditions later in life” [35]. The information gleaned from assessing youth and adult substance use and ACEs data can lead to new insights and policies for implementing prevention programs targeted to address complex mental and physical health needs.

Indeed, funding decisions, use of evidence-based programs, and health services are enacted at the larger systems level. Often, the policies develop from state, county, city, or coalition level strategies around the development and implementation of three-to-five-year health improvement plans. Such plans rely on needs assessment data to develop a strategy and garner stakeholder buy-in. Including youth and adult substance use and ACEs data collection and cross-data analyses in addressing the role of ACEs in allocating resources for substance use prevention programs allows decision-makers to accomplish the following:

- Select programs that are targeted to specific age and ethnic population groups. Research has shown that targeted programming impacts program acceptance and successful outcomes [36, 37].
- Select evidence-based programs to the extent that matches can be found between the target audiences and programs; at a minimum, all programs should demonstrate an evidence-informed framework and data results that show positive outcomes.
- Select trauma-specific services and/or infuse a trauma-informed component into all prevention, intervention, and treatment programs and policies.
- Address the co-morbidity of substance use and trauma as a major consideration in program and policy implementation.
- Adopt statewide policies that support the implementation of targeted prevention, intervention, and treatment programs and practices.
- Integrate prevention plans, programs, and policies across a variety of system-level and funding sources.

## Limitations

This study made unique contributions by examining the relationship between ACEs and substance use outcomes between adolescents and adults. Despite the contributions of the current study, this study has several limitations. First, this study used cross-sectional data, and therefore, causality cannot be determined. Second, because the data are based on self-reports of drug misuse, participants may be subject to underreporting and/or recall bias. In addition, the substance use measures were all dummy coded as use or not use, which for future surveys, it would be better to collect the specific days or amount of using substances (as a continuous measure). Third, the results of this study can only be generalized to participants living in Arizona, and the results should not be generalized to the youth and adults in the United States. Fourth, the measurement of ACEs in this study does not include items measuring child abuse or neglect due to limitations in the Arizona Revised Statutes for the AYS about not allowing questions on depression, religiosity, sex, or morality in order to be exempt from parental consent for the survey; BRFSS did ask 10 ACEs questions but only similar questions across the two surveys were analyzed. Due to this limitation, the findings may be conservatively estimating the effect of ACEs on substance use as major aspects of ACEs were left out.

## Conclusion

It was determined as feasible and methodologically possible to conduct comparisons using similar analyses on similar variables across different youth and adult datasets to produce meaningful results regarding relationships that existed between substance use and ACEs. Using local research can be essential to sound decision-making, and this methodology is a generalizable approach for conducting comparative analyses. The results in this study showed that the findings from the youth (AYS) and adult (BRFSS) datasets were consistent: users having an ACEs score of 4 to 6 had a positive association with more substance use. Comparing these data also showed that the need for evidence-based prevention and intervention programs will continue in the foreseeable future as these youth become adults.

The results from the identified relationships between substance use and trauma data should be used to inform prevention, intervention, and treatment programs and policies in Arizona and could be modeled elsewhere. Conducting data analysis using both youth and adult data on substance use and ACEs followed by data-driven decision-making provides a process to address needs, resources, practices, and policies that impact substance use outcomes around systems and individuals regarding substance use and trauma programs, policies, and priorities.

## Data Availability

The dataset of AYS is available on ACJC website at https://www.azcjc.gov/Programs/Statistical-Analysis-Center/Arizona-Youth-Survey. Application for access can be made through the Statistical Analysis Center of ACJC. The center office will review the application. Requests can be sent to the coordinator of the Statistical Analysis Center of ACJC using the following email: AYS@azcjc.gov. The BRFSS datasets are public use data, and researchers can directly download the survey data on CDC website at https://www.cdc.gov/brfss/data_documentation/index.htm.

## Abbreviations

ACEs: Adverse childhood experiences
AYS: Arizona Youth Survey
BRFSS: Behavioral Risk Factor Surveillance System
ADHS: Arizona Department of Health Services
CDC: Centers for Disease Control
SAMHSA: Substance Abuse and Mental Health Services Administration
YRBS: Youth Risk Behavior Survey
UHF: United Health Foundation
